# Feasibility and Acceptability of a Text Message-Based Smoking Cessation Program for Young Adults in Lima, Peru: Pilot Study

**DOI:** 10.2196/mhealth.7532

**Published:** 2017-08-04

**Authors:** Dora Blitchtein-Winicki, Karine Zevallos, M Reuven Samolski, David Requena, Chaska Velarde, Patricia Briceño, Marina Piazza, Michele L Ybarra

**Affiliations:** ^1^ Mental Health, Alcohol and Drug Unit Public Health Department Universidad Peruana Cayetano Heredia Lima Peru; ^2^ Executive Office of Research Peruvian National Institute of Health Lima Peru; ^3^ Centro de Investigación en Enfermedades Tropicales “Maxime Kuczynski” Peruvian National Institute of Health Loreto Peru; ^4^ Peruvian National Institute of Health Lima Peru; ^5^ Center for Innovative Public Health Research San Clemente, CA United States

**Keywords:** Pilot Projects, Text Messaging, Smoking Cessation, Young Adult, Cognitive Therapy, Feasibility Studies, Latinos

## Abstract

**Background:**

In Peru’s urban communities, tobacco smoking generally starts during adolescence and smoking prevalence is highest among young adults. Each year, many attempt to quit, but access to smoking cessation programs is limited. Evidence-based text messaging smoking cessation programs are an alternative that has been successfully implemented in high-income countries, but not yet in middle- and low-income countries with limited tobacco control policies.

**Objective:**

The objective was to assess the feasibility and acceptability of an short message service (SMS) text message-based cognitive behavioral smoking cessation program for young adults in Lima, Peru.

**Methods:**

Recruitment included using flyers and social media ads to direct young adults interested in quitting smoking to a website where interested participants completed a Google Drive survey. Inclusion criteria were being between ages 18 and 25 years, smoking at least four cigarettes per day at least 6 days per week, willing to quit in the next 30 days, owning a mobile phone, using SMS text messaging at least once in past year, and residing in Lima. Participants joined one of three phases: (1) focus groups and in-depth interviews whose feedback was used to develop the SMS text messages, (2) validating the SMS text messages, and (3) a pilot of the SMS text message-based smoking cessation program to test its feasibility and acceptability among young adults in Lima. The outcome measures included adherence to the SMS text message-based program, acceptability of content, and smoking abstinence self-report on days 2, 7, and 30 after quitting.

**Results:**

Of 639 participants who completed initial online surveys, 42 met the inclusion criteria and 35 agreed to participate (focus groups and interviews: n=12; validate SMS text messages: n=8; program pilot: n=15). Common quit practices and beliefs emerged from participants in the focus groups and interviews informed the content, tone, and delivery schedule of the messages used in the SMS text message smoking cessation program. A small randomized controlled pilot trial was performed to test the program’s feasibility and acceptability; nine smokers were assigned to the SMS text message smoking cessation program and six to a SMS text message nutrition program. Participant retention was high: 93% (14/15) remained until day 30 after quit day. In all, 56% of participants (5/9) in the SMS text message smoking cessation program reported remaining smoke-free until day 30 after quit day and 17% of participants (1/6) in the SMS text message nutrition program reported remaining smoke-free during the entire program. The 14 participants who completed the pilot reported that they received valuable health information and approved the delivery schedule of the SMS text messages.

**Conclusions:**

This study provides initial evidence that a SMS text message smoking cessation program is feasible and acceptable for young adults residing in Lima.

## Introduction

Smoking tobacco and exposure to particulate matter and carcinogenic agents in tobacco smoke have detrimental effects on health [1,2] leading to a greater incidence of cancer; cardiac, cerebrovascular, and respiratory failure; eye and skin damage; and fertility problems [1]. Smoking is highly addictive due to pharmacological [3] and psychological factors, making it difficult to quit [4,5]. Studies have demonstrated that interventions for smoking cessation can be effective [6,7] at reducing morbidity and mortality related to tobacco use [6,8-10].

Increased awareness of tobacco-related morbidity and mortality has led many countries to implement policies, control measures, and interventions for smoking cessation [5,7], which have helped to reduce the prevalence of smoking in developed countries [11]. However, developing countries such as Chile, Costa Rica, Jamaica, Suriname, and Peru [12] have maintained a high prevalence of tobacco smoking: approximately 20% to 30% in men and 10% to 20% in women [13]. In urban areas of Peru, tobacco smoking begins between 12 and 18 years. Young adults have a higher prevalence rate. In 2013, 3 in 10 individuals aged between 19 and 28 years in Lima smoked at least one cigarette during the year compared to 1 in 10 individuals aged between 12 and 65 years [14]. Between 6 and 7 of 10 adolescents in urban areas of Peru who smoke tobacco have tried to quit, most of whom were not successful [15].

Mobile phones can be effective public health interventions due to their ubiquity and relative ease of use. Delivering text messages is program-initiated (one-way) and proactive, reaching the user wherever he or she happens to be, with minimal barriers (the mobile phone notifies the user when a short message service [SMS] text message is received and SMS text messages may automatically appear on the screen) [16-19]. In addition, using cell phones avoids the high cost of in-person interactions with health personnel and facilitates the efficient collection and processing of information. Messages can be adapted based on the user’s characteristics and delivered at any time of day [20,21]. It is also cost-effective and can reach a large number of people in a large geographical area [22].

Most people in Peru own mobile phones. In 2014, there were approximately 104 mobile phones for every 100 inhabitants according to the Ministry of Communications and Transportation [23]. In particular, young people are more prone to carry mobile phones and use text messages. In Peru, the use of mobile technology has demonstrated great potential as a cost-effective health strategy to improve access to health care [24].

There is evidence that SMS text messaging programs using cognitive behavioral therapy (CBT) for smoking cessation are effective, increasing its likelihood of success by 35% in comparison with control groups [17,25-27]. Its success rate is similar to other interventions, such as helpline services to quit smoking, and face-to-face cognitive behavioral and cognitive therapy and counseling [25,28], with the additional benefit of being delivered at a lower cost [17]. Compared with other treatment methods, there is evidence that SMS text message-only interventions affect 6-month cessation outcomes similarly to other effective treatments [26,29-31]. Likewise, a meta-analysis that included 20 manuscripts with 22 interventions showed that SMS text message cognitive behavioral smoking cessation programs can be an effective strategy to help individuals stop smoking [27].

Intervention strategies for smoking cessation should consider its target population [30,32]. Attitudes and behaviors related to tobacco use are often dependent on factors such as gender, race, and age, among others. For example, most Latinos prefer nonpharmacological methods to quit smoking (eg, replacement of nicotine, bupropion, varenicline) [33,34], whereas a lower proportion of young people decide to use these therapies compared with other age groups [35].

### Theoretical Frameworks

The theoretical frameworks that guided the development of the SMS text message cognitive behavioral smoking cessation program were the transtheoretical model, CBT, increasing self-efficacy, and relapse prevention. As defined by the transtheoretical model, behavior change is a process over time and involves progression through a series of stages: precontemplation, contemplation, preparation, action, and maintenance [36,37]. Program content was based on CBT and increasing self-efficacy, and program components identified in smoking cessation telephone-based counseling (quitlines) [4,30,38-41]. CBT focuses on cognitive restructuring, education, self-monitoring, and practical coping strategies aimed at successful smoking cessation. CBT implies that mental rules guide behavior, such that applying illogical beliefs (cognitive processes) lead to dysfunctional behavior (behavioral actions). To acquire the capability to quit smoking, individuals need to recognize, understand, and change their behavior patterns. Increasing self-efficacy was also considered a key element in developing the SMS text message cognitive behavioral program content [42-44] because it includes constructs such as perceived temptations and self-regulatory strategies increases relapse prevention [45] and low self-efficacy may undermine the ability to maintain or initiate efforts to cope with high-risk situations [46-48]. Relapse prevention is a cognitive behavioral approach to relapse with the goal of identifying and preventing high-risk situations; in the case of smoking cessation, preventing smoking after a period of abstinence [49,50]. Relapse prevention includes adapting coping responses to life stressors and risky situations by minimizing exposure to cues. For example, understanding that discomfort will reach a peak and then subside, encouraging patience to wait out the urge, using nicotine replacement treatment (NRT), and seeking health professional advice about other pharmacological evidence-based treatments [51-53].

The objective of this study is to evaluate the feasibility and acceptability of an SMS text message cognitive behavioral program for smoking cessation among young people aged 18 to 25 years who live in Lima, Peru. In this study, we report on feasibility and acceptability of an SMS text message cognitive behavioral program for smoking cessation and develop initial content and methodology in preparation for a randomized controlled trial (RCT).

## Methods

### Recruitment

**Figure 1:**
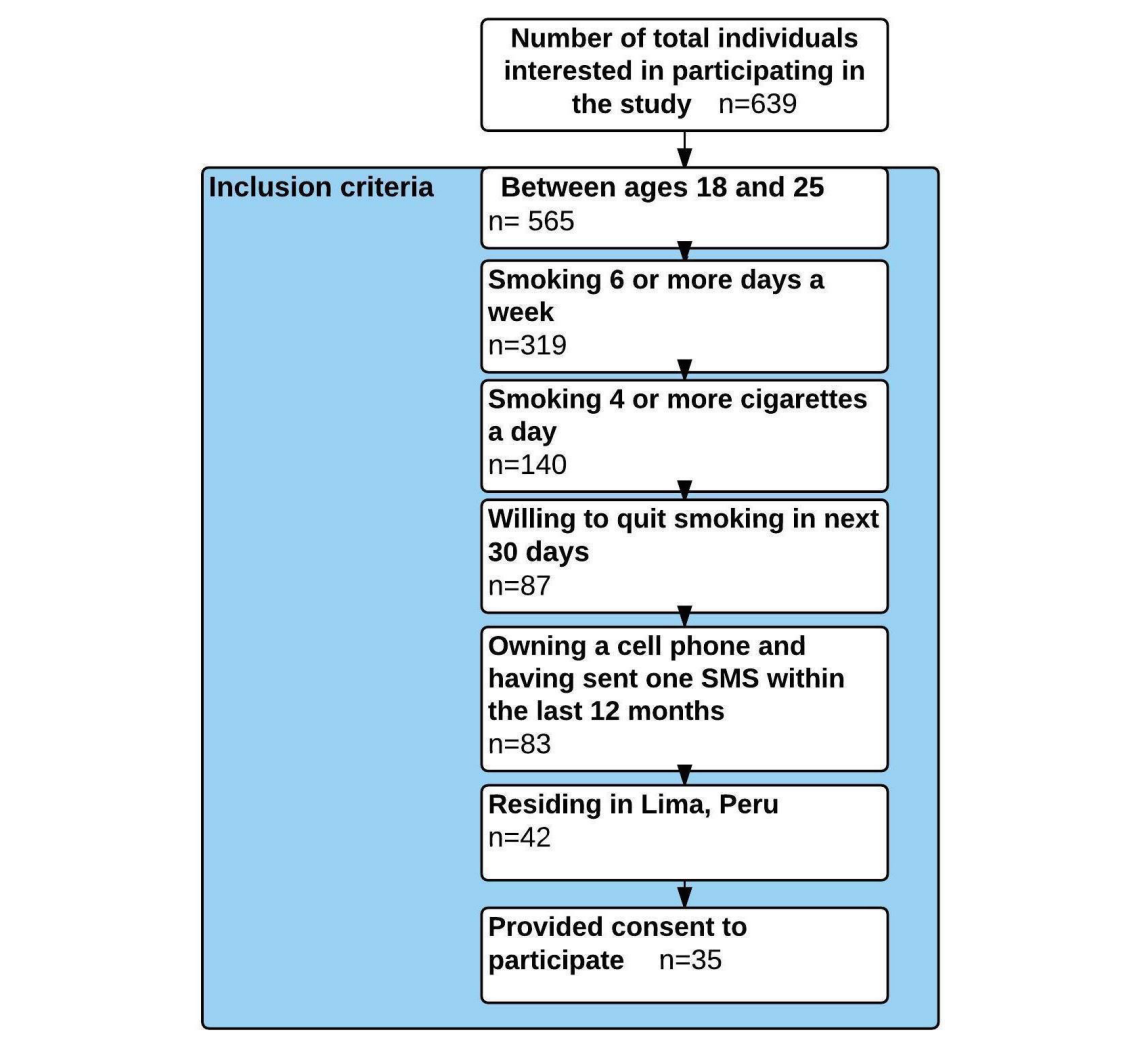
Flowchart of individuals included in the study based on inclusion criteria.

Participants were recruited through advertisements on Facebook and Google, a Facebook fan page, and flyers in locations where young people frequent (eg, cafeterias, stores, pubs, technical schools, universities, near public transportation), which directed them to a website with smoking cessation information. Participants had to be interested in quitting smoking and seeking help to quit. Those interested in participating completed an online eligibility questionnaire on the website, which was hosted on Google Drive. Eligible participants were residents of Lima, between ages 18 and 25 years, smoked four or more cigarettes daily, had their own cell phone, and sent and received at least one SMS text message in the previous 12 months. We measured intention to quit by asking: “Are you seriously thinking of quitting smoking?” Response options were (1) “No, not thinking about quitting,” (2) “Sometime, but not within the next 6 months,” (3) “Yes, within the next 6 months,” and (4) “Yes, within the next 30 days.” Only those who selected the fourth option were eligible. Those who met the eligibility criteria were contacted by a member of the research team who provided a description of the study, answered questions, and asked participants to provide informed consent. Recruitment continued during the entirety of the study. Figure 1 shows the eligibility criteria used to evaluate individuals interested in participating in the study. Content development and technical development are discussed in further detail subsequently.

All participants who met the inclusion criteria were included in only one of the following study phases, which were conducted consecutively:

1. Phase A: adapting SMS smoking cessation and nutrition program content with focus groups and in-depth interviews;
2. Phase B: individual sessions to validate SMS text messages with young adults; and
3. Phase C: SMS text message smoking cessation program pilot where participants were randomly assigned to the SMS text message smoking cessation program (intervention) or the SMS text message nutritional program (control).

**Figure 2:**
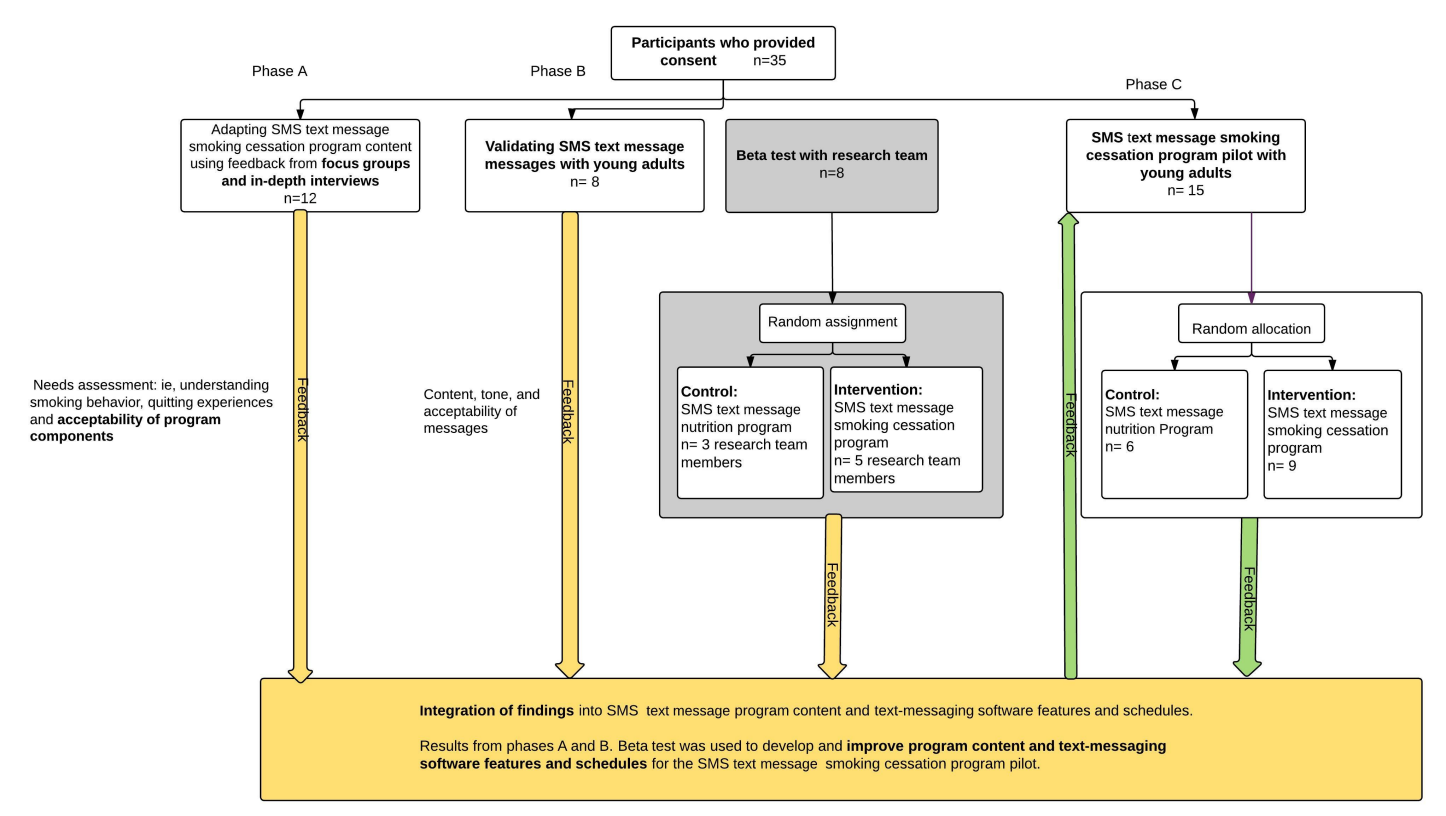
Program outline and participation in different phases of the study.

Figure 2 describes the main components of the study and the number of participants in each phase. All participants provided informed consent. Prior to phase C, the smoking cessation program was piloted with members of the research team. The pilot study was conducted between July 2013 and November 2014. Recruitment lasted 10 months.

### Content Development

#### General Characteristics of the SMS Text Message Smoking Cessation Program (Intervention)

The smoking cessation SMS text message program content consisted of tailored messages and pathways based on individual assessment [54]. Program content was tailored to create individualized intervention strategies according to gender, stage of quitting (prequit, quit, relapse), and smoking status at different stages of the study. Likewise, participants received bidirectional messages, which allowed them to provide self-reported smoking cessation data on days 2, 7, and 30 after quit date. Participants received tailored sets of messages depending on their responses, which were sent automatically using algorithms.

**Figure 3:**
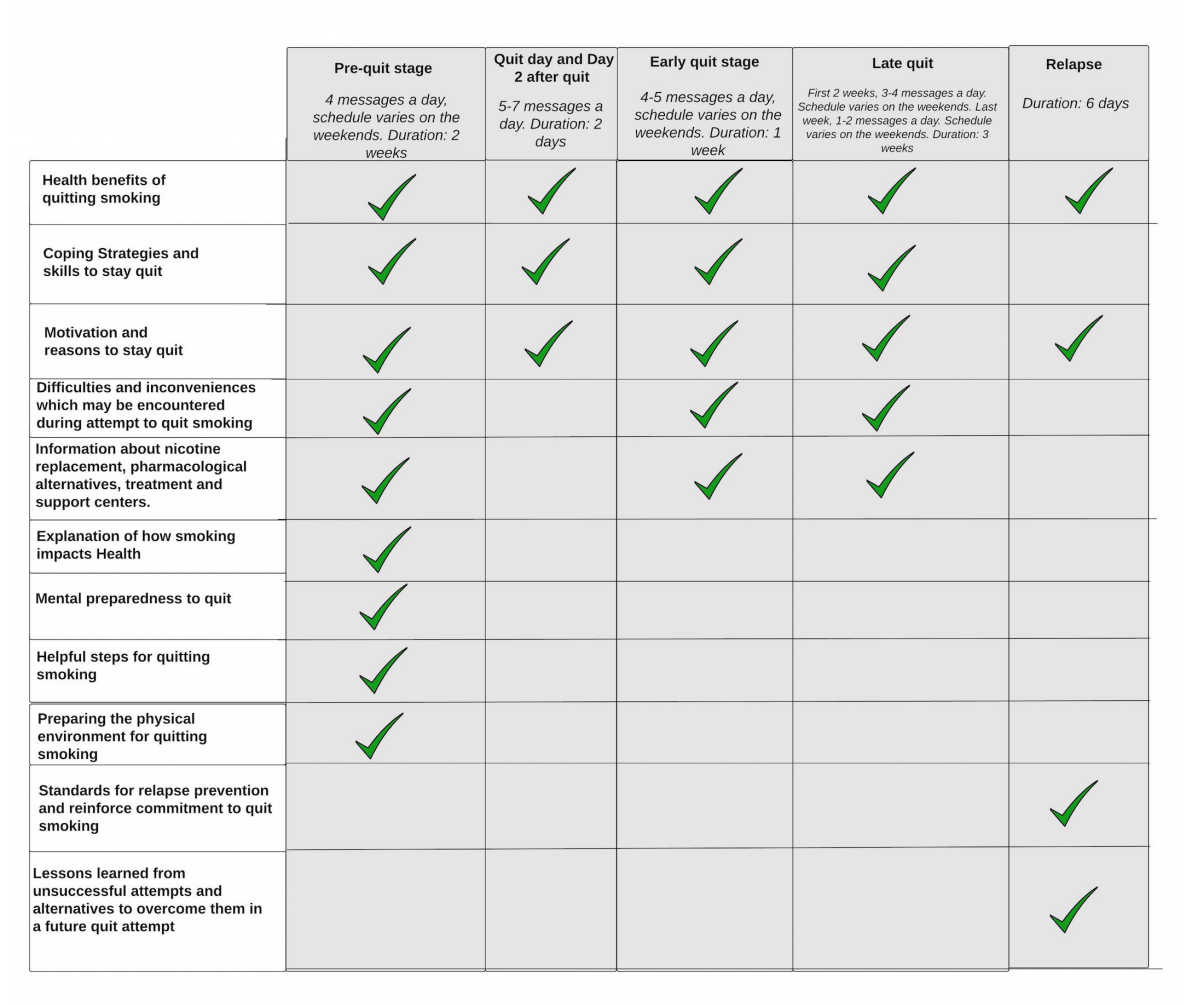
Types of information included in each stage of the SMS text message smoking cessation program.

Content was developed based on the theoretical frameworks described previously, and participant feedback through focus groups and in-depth interviews to validate the SMS text messages with the target population. Examples of the type of information included in the SMS text message smoking cessation program are shown in Figure 3.

#### Examples of Types of Information Included in Different Stages of the SMS Text Message Cognitive Behavioral Smoking Cessation Program

##### Prequit Stage

1. Organizing a plan to quit smoking: setting a date; informing family and friends; asking for their support; identifying situations, triggers, and risks (eg weekends, friends, and social activities), and alternatives to cope with or avoid them; identifying social support resources; planning to reward oneself for achieving short-term smoking cessation goals; and educating oneself about how to avoid nicotine withdrawal symptoms.
2. Preparing the physical environment for quitting smoking: removing cigarettes and objects related to smoking.
3. Preparing mentally to quit smoking: for example, debunking myths such as “smoking is ok as long as I engage in other healthy behaviors” or “e-cigarettes are the most effective method to quit smoking.” Messages that promote self-confidence and identifies benefits and reasons to quit and stay tobacco free.
4. Motivational content that reinforces commitment to quit.

##### Quit Day and Day 2 After Quit

1. Coping strategies and skills to stay quit: activities and suggestions that help to stay quit and meet short-term quit goals.
2. Motivation: celebrate successes and reinforce commitment to quit.

##### Early Quit Stage

1. Coping strategies and skills to stay quit: reminders of reasons for quitting; engaging in other enjoyable activities; how to cope with withdrawal side effects; helpful tips, such as keeping hydrated; and capitalizing on prior successful actions and strategies to stay quit.
2. Difficulties and inconveniences: for instance, how to cope with common withdrawal symptoms, what to expect and how to face them, and when they are expected to appear.

##### Late Quit

1. Coping strategies and skills to stay quit: address concerns such as gaining weight and how to manage stress without smoking, how to identify and handle relapse triggers, and how to deal with cravings.
2. Motivation to stay quit: planning activities to celebrate each milestone achieved, preparing to continue, perceiving self as a nonsmoker, and continuing setting short-term goals.
3. Difficulties and inconveniences: for instance, recognizing gains in health and information about withdrawal symptoms and timeline.

##### Relapse

1. Standards for relapse prevention and commitment to quit smoking: positive reminders of achievements; information about how successfully quitting smoking may require several attempts, but is achievable; reinforcing messages about benefits of quitting and motivations and steps for continuing to try quitting.
2. Lessons learned from an unsuccessful attempt and alternatives to overcome them in a future quit attempt: identify reasons for relapse and employ prevention strategies in next quit attempt, and encouragement to try quitting smoking again within a short period of time (one month).

### General Characteristics of the SMS Text Message Nutrition Program (Control)

The nutrition text messages were designed to motivate healthy eating and physical activity in order to achieve health benefits. Messages included increasing vegetable intake, decreasing sweetened beverage consumption, and improving eating behaviors and physical activity.

### Focus Groups and In-Depth Interviews

We held two focus groups and four in-depth interviews with 12 participants with the aim to provide insight into young adult smoking behavior, SMS text message-related behavior and preferences, experiences with quitting smoking, and perceived reasons for prior unsuccessful smoking attempts. The themes explored included participants’ initial smoking experiences, smoking practices, smoking-related perceptions, reasons for continuing smoking, previous attempts to quit smoking, reasons for deciding to quit, the influence of close friends and family on quitting, perceived consequences of smoking, strategies and activities to stay quit, knowledge and prior use of alternative tobacco products, use of mobile phones for text messaging, acceptability of using SMS text messages to quit smoking, and preferences regarding the type, frequency, and content of SMS text messages they would like to receive. Their answers were audiotaped, transcribed, and analyzed and considered in the adaptation and development of the program content.

### Validating SMS Text Messages With Young Adults

Once we had a final pool of messages, eight participants were recruited to review a set of messages from the proposed SMS text message smoking cessation program. A member of the research team met for 1.5 to 2 hours with each participant individually and explained the purpose of the SMS text message program, and the role and importance of using messages in the program. Each participant was given a list of 60 messages to review between the initial meeting and a follow-up meeting 7 to 10 days later, using a guide provided by the researchers. During the follow-up meeting, they were requested to provide feedback on the messages. At least two participants were asked to review each SMS text message to ensure that all the messages had been reviewed. Each participant received messages from all the stages in the program (prequit, quit day, early quit, late quit, and relapse). Participants were asked to provide feedback on the following: was the message understandable, how did they perceive the tone of the message, was the message phrased in a way that young adults could relate, and did the messages provide relevant information according to the participant’s smoking status. Four research team members modified the SMS text messages based on participant feedback.

Participants received a code of prepaid text message credit equivalent to US $6 after each face-to-face meeting (two meetings per participant).

### Technological Development

The automated text messaging system was developed using the following components: list of SMS text messages for both programs, list of participants’ cellphone numbers, and randomizing methodology and tailoring criteria for the SMS text message smoking cessation program and the SMS text message nutrition program. The system used a script written in Python 2.7 in the app SMS Scheduler [55] to manage, schedule, and send SMS messages. The system was configured on a Samsung Galaxy S5 mini with Android 4.4 (KitKat).

**Figure 4:**
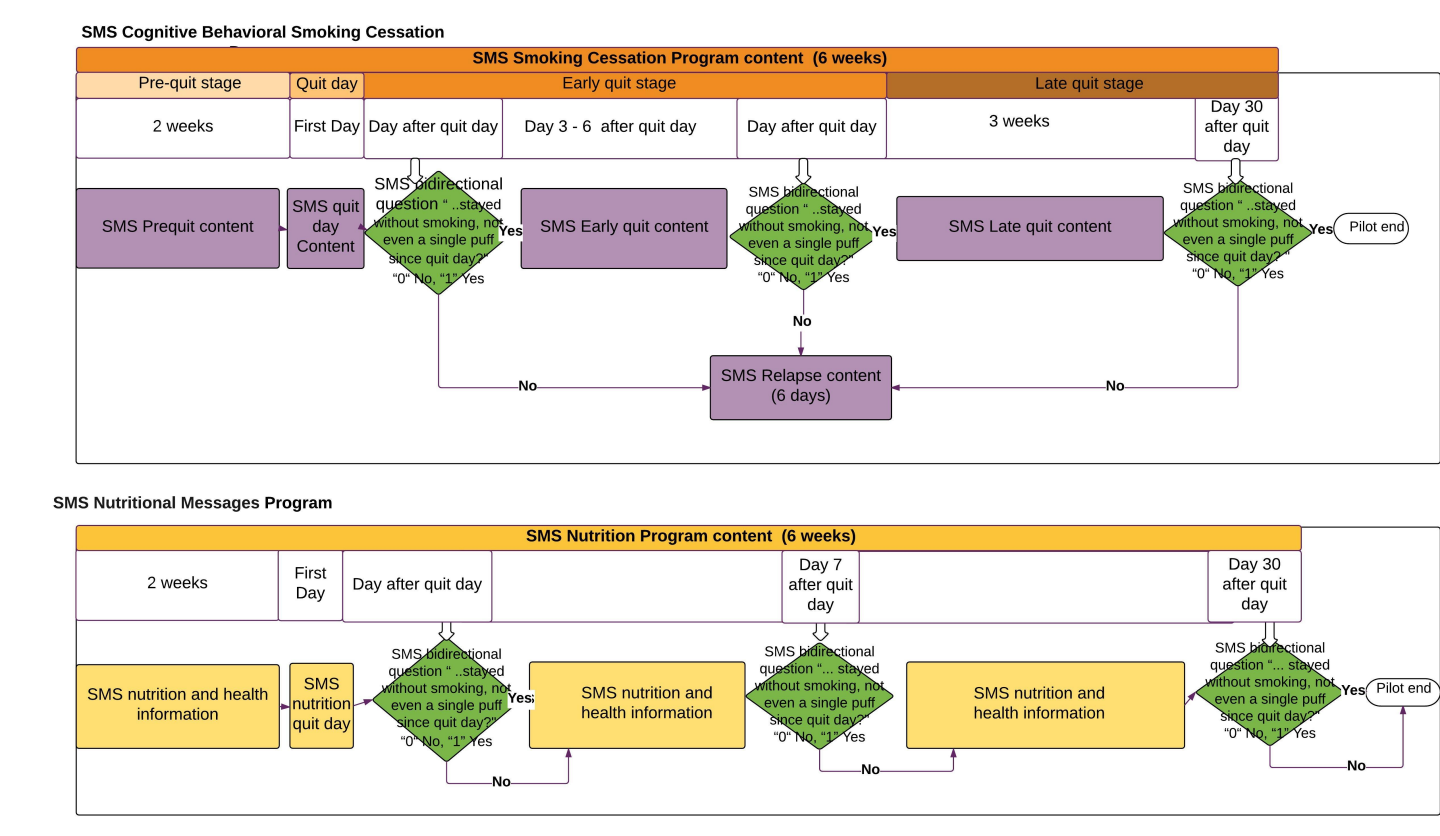
Flowchart of the SMS text message smoking cessation program and the SMS text message nutrition program pathways.

On days 2, 7, and 30 after the participant’s quit day, a message was sent to participants asking them about their ability to remain smoke-free. Participants answered 1 (“yes”) or 0 (“no”), which the system registered and used to automatically assign the next set of messages to be received by the participant in the SMS text message smoking cessation program. In the SMS text message nutrition program, participants received the same set of messages regardless of their response, as shown in Figure 4.

### Beta Test of the SMS Text Message Smoking Cessation Program With the Research Team

Before piloting the SMS text message smoking cessation program with the participants, it was tested by members of the research team over 6 weeks to troubleshoot any technological difficulties. Three research team members received the SMS text message nutrition program (control) and five members the SMS smoking cessation program (intervention). During the beta test, various difficulties were addressed: the software was corrected to include letters and symbols used in the Spanish language; difficulties with scheduled SMS text message delivery were solved by sending SMS text messages separately and restricting simultaneous messages; due to constant changes in cell phone ownership, the research team frequently updated the participants’ contact information and encouraged participants to take advantage of mobile numerical portability (MNP), which allows users to retain their mobile telephone numbers independent of changes in cell phone or mobile network carrier.

#### Piloting the SMS Text Message Smoking Cessation Program with Young Adults

Fifteen participants were recruited to test the feasibility and acceptability of the SMS text message smoking cessation program. The participants were randomly assigned to the SMS text message smoking cessation program (nine participants) or the nutrition program (six participants). Only two women were recruited for the pilot; one woman was assigned to each arm through randomized assignment. Every week, participants sent feedback about the content and timing of the SMS text messages received. Feedback received was used to measure acceptability of the program among participants.

The outcome—successfully quitting on days 2, 7, and 30 after quit day—was measured using self-reports of smoking cessation for both arms of the study. We sent an SMS bidirectional question on days 2, 7, and 30 after the participant’s quit day, asking “Have you stayed tobacco free (not even a single puff) since the day you quit?” If yes, the participant answered “1” and if no, the answer was “0.” The answer allowed the program to assign the next set of messages that the participant received as shown in Figure 4. The research assistant called to remind the participant to answer the SMS text question the same day the bidirectional question was sent. This approach was effective for almost all the participants, except for one who left the country during the study.

The participants were asked to provide feedback weekly to minimize recall bias. They sent their comments through SMS text message and they received the equivalent of US $3 in credit for mobile calls and SMS text message through a code sent to them by SMS text message to compensate for the cost of sending SMS text messages related to the program.

To assess the feasibility we considered issues related to ease of implementation of the SMS text message pilot study, and any technical problems and issues which arose from taking part in the intervention or control arm as other SMS text message studies have previously measured [16,20,56,57].

We included the following measures:

1. Recruitment rate: ability to recruit our target sample size of 75 participants from Peru (of which 40 were residents of Lima) in the time allotted for the project, which was 10 months.
2. Retention rates: measures included (1) within the SMS text message smoking cessation program, whether participants responded to queries about their smoking status on days 2, 7, and 30 after quit day, and (2) in the research, to be able to achieve the follow-up rates necessary to conduct a RCT at day 30 after quit day (93% of 15 participants).
3. Performance of the software that delivers the program’s text messages (eg, successful delivery of messages to all designed mobile phone carriers).

To assess the acceptability of the program, we considered consent and response rates during the entirety of the study, and comments and feedback about their experiences with the study [16,57,58].

We included the following measures: participant responses to the text messages, feedback from participants, the number of participants who asked to be removed from the program during the intervention phase, and a series of questions about the program’s characteristics and likability, administered one month after the quit day.

This pilot study was approved by the institutional review boards of Michigan State University and Universidad Peruana Cayetano Heredia.

### Statistical Analysis

All data during the SMS text message smoking cessation program pilot was entered into a Microsoft Excel 2011 database. Data was cleaned and checked for disparities before and after the data entry. Descriptive statistics (percentage, mean, median, standard deviation) were used to describe demographic, smoking-related characteristics, SMS text message use, nicotine dependence, and smoking cessation self-report outcome of respondents. Descriptive analyses were performed using STATA (12.0 version). Nicotine dependence was classified using the Fagerström Test of Nicotine Dependence (FTND) score and used the following categories: very low/none, low, medium, and high [3].

## Results

### Participants

A total of 639 individuals answered our online screening survey; 565 met the inclusion age criteria (18-25 years), 319 reported having smoked 6 to 7 days a week (frequency), 140 smoked four or more cigarettes a day (intensity), 87 had decided to quit smoking in the next 30 days, 46 lived in Lima, and two did not have a mobile phone (Table 1). Forty-two participants had sent at least one SMS text message in the previous 12 months of which 35 consented to participate in the pilot study: focus groups and in-depth interviews (n=12), young adult participants to validate messages (n=8), and the validation of the SMS text message smoking cessation program (n=15). We recruited during the entirely of the study using paid Facebook ads to increase recruitment rates, recruiting 70 individuals with the inclusion criteria at the national level, of which 42 were Lima residents.

**Table 1:** Characteristics of participants in the SMS text message smoking cessation program pilot.

Characteristics of participants		Smoking cessation (intervention), n (%) (n=9)	Nutrition (control), n (%) (n=6)	Total, n (%) (N=15)
**Age**				
	18-19	1 (11)	2 (33)	3 (20)
	20-21	4 (44)	3 (50)	7 (47)
	22-23	4 (44)	1 (17)	5 (33)
**Gender**				
	Female	1 (11)	1 (17)	2 (13)
	Male	8 (89)	5 (83)	13 (87)
**Education level**				
	Secondary education completed	1 (11)	0 (0)	1 (7)
	Technical studies not completed	1 (11)	2 (33)	3 (20)
	University education not completed	6 (67)	4 (67)	10 (67)
	University completed	1 (11)	0 (0)	1 (7)
**Carrier**				
	Claro	7 (78)	4 (67)	11 (73)
	Movistar	2 (22)	1 (17)	3 (20)
	Entel	0 (0)	1 (17)	1 (7)
**Service plan**				
	Prepaid	2 (22)	0 (0)	2 (13)
	Postpaid	7 (78)	6 (100)	13 (87)
**Average number of SMS text messages received per day at the beginning of the study**				
	≤5	5 (56)	3 (50)	8 (53)
	6-10	2 (22)	2 (33)	4 (27)
	≥11	2 (22)	1 (17)	3 (20)
**When do you check your SMS text messages?**				
	As soon as it arrives	8 (89)	5 (83)	13 (87)
	During the afternoon	0 (0)	1 (17)	1 (7)
	At night	1 (11)	0 (0)	1 (7)
**Dependency level (FTND scale)**				
	Very low	5 (56)	4 (67)	9 (60)
	Low	2 (22)	0 (0)	2 (13)
	Mild	1 (11)	2 (33)	3 (20)
	High	1 (11)	0 (0)	1 (7)
**Do you live with other people who smoke?**				
	No	5 (56)	2 (33)	7 (47)
	Yes	4 (44)	4 (67)	8 (53)
**Have you had problems with alcohol and other drugs?**				
	No	5 (56)	4 (67)	9 (60)
	Yes	4 (44)	2 (33)	6 (40)
**Do you have a medical problem?**				
	No	7 (78)	4 (67)	11 (73)
	Yes	2 (22)	2 (33)	4 (27)
**Medical problem**				
	Asthma	0 (0)	1 (17)	1 (7)
	Back pain	1 (11)	0 (0)	1 (7)
	Prediabetes	1 (11)	0 (0)	1 (7)
	Renal inflammation nephritis	0 (0)	1 (17)	1 (7)
Have you tried to quit smoking before? (yes)		9 (100)	6 (100)	15 (100)
**Number of previous quit attempts**				
	1	1 (11)	1 (17)	2 (13)
	2	2 (22)	2 (33)	4 (27)
	3	4 (44)	1 (17)	5 (33)
	4	2 (22)	2 (33)	4 (27)
**Have you used some kind of aid when you tried to quit?**				
	No	5 (56)	5 (83)	10 (67)
	Yes	4 (44)	1 (17)	5 (33)
**What type of aid?**				
	Chewing gum	1 (11)	0 (0)	1 (7)
	E-cigarette	2 (22)	1 (17)	3 (20)
	Book	1 (11)	0 (0)	1 (7)
**What’s the longest time you’ve been smoke-free (not even a puff), from any prior quit attempt?**				
	1 day	0 (0)	1 (17)	1 (7)
	3 days	3 (33)	0 (0)	3 (20)
	1 week	1 (11)	2 (33)	3 (20)
	2 weeks	0 (0)	1 (17)	1 (7)
	3 weeks	1 (11)	0 (0)	1 (7)
	1 month	1 (11)	0 (0)	1 (7)
	2 months	0 (0)	1 (17)	1 (7)
	3 months	3 (33)	1 (17)	4 (27)
**Quit report “not even a puff” on day 7 after quit^a^**				
	No (relapse)	4 (44)	2 (33)	6 (40)
	Yes	5 (56)	4 (67)	9 (60)
**Quit report “not even a puff” on day 7 after quit^a^**				
	No (relapse)	0 (0)	3 (50)	3 (20)
	Yes	5 (56)	1 (17)	6 (40)
**Quit report “not even a puff” on day 30 after quit day^a^**				
	No (relapse)	0 (0)	0 (0)	0 (0)
	Yes	5 (56)	1 (17)	6 (40)

^a^ Participants answered 0=“no” or 1=“yes.” Each participant could only answer “no” once. On answering no, they were sent messages from the relapse stage for 6 days.

### Adapting SMS Text Message Smoking Cessation and Nutrition Program Content with Focus Groups and in-Depth Interviews

Findings from the focus group discussions and in-depth interviews demonstrated that the majority of the participants (n=12) who wanted to quit smoking did not have a plan. Most had tried previously but were unsuccessful. What they least liked about smoking was the smell, increased probability of having wrinkles and yellow teeth, and the shortness of breath during regular activities such as climbing stairs. They were unfamiliar with evidence-based alternatives and were not be willing to take medication. They were willing to try nicotine patches, but they did not know where to get them; cost was also a barrier. Many considered the e-cigarette an easily accessible alternative to cigarettes and knew acquaintances who had used it to quit.

The biggest obstacle to quitting was smoking triggers during the weekends: meeting friends who smoke, being at bars and parties, and being around people who smoke. One of the most common reasons for individuals to not quit smoking was the fear of gaining weight.

From the results of the focus groups, messages were added or modified to address participant concerns and triggers (see Multimedia Appendix 1). For instance, one theme that emerged was the idea that healthy behaviors compensated for the negative health effects of smoking. Another main concern about quitting was weight gain. We included messages related to the following: including exercise in their plan to quit, consuming low-calorie foods, drinking lots of water, and focusing all their efforts on quitting (only after they successfully quit smoking should they focus on other goals).

### Validating SMS Text Messages with Young Adults

Once we had a final pool of messages, we recruited eight participants to validate the messages. The group read and provided feedback on each text message. Recommendations included rewording the messages, expressions and tone as if a peer had sent the messages, sending different messages for men and women, personalizing certain messages by including their name, and sending messages during certain hours or days of the week (eg, sending messages on the weekends about alternative enjoyable activities instead of going to a bar with friends). At least two participants reviewed each message. Suggestions were reviewed by four research team members and were used to improve the program messages and delivery schedule. Examples of the messages are found in Multimedia Appendix 2.

### Beta Test With Research Team

Overall the software program and messaging infrastructure (eg, mobile phone and server) were reliable. Text messages were sent to eight research team members during the beta test. Certain issues arose such as the initial inability to use accented letters and Latin characters, inability to send multiple messages simultaneously, and frequent changes in mobile network operators. We identified strategies to overcome these barriers, such as finding an alternative to include accented letters and Latin characters, and asked participants to take advantage of MNP when their mobile phone was lost or stolen. MNP allows an individual to retain the same phone number regardless of changing from one mobile network carrier to another was an advantage.

### SMS Text Message Smoking Cessation Program Pilot

Of the 15 young adults who provided consent to participate in the SMS text message smoking cessation program, more than 87% (13/15) were male, and all reported having attempted to quit smoking in the past on at least two previous occasions (Table 1). Three young adults (20%) used an e-cigarette as an aid to quit smoking in previous attempts; 53% (8/15) lived with other smokers and 40% (6/15) reported previous consumption of alcohol and/or drugs. The level of nicotine dependence was measured with the FTND. Based on the FTND score, nine (60%) had very low dependence, two (13%) had low, three (20%) had mild, and one (7%) had high.

The intervention retention rate was high: 14 of 15 participants (93%) remained until day 30 after quit day. One participant traveled out of the country and could not continue. Fourteen participants were contacted weekly during their participation and on day 30 after quit day. We called all the participants at the end of the same day the question was sent. If they did not answer the question, we reminded them to answer the text question, on which the participant was assigned to the corresponding pathway.

All nine individuals assigned to the intervention to quit smoking completed the program. Length of participation in the program was dependent on their answers regarding quitting smoking; for instance, if the participant responded “no” to “Have you stayed tobacco free (not even a puff) since the day you quit?” then they were assigned to receive messages with content from the relapse stage for 6 days. The control arm was completed by five of six (83%) of the participants.

All participants in the intervention group (n=9) stated that they received valuable information about health issues, treatment resources, NRT, and strategies to avoid smoking. Furthermore, 78% (7/9) reported that the messages were motivating and easy to understand, 89% (8/9) reported that they would recommend the program to others, 78% (7/9) agreed with the number of messages sent during the duration of the program, and 100% (9/9) agreed with the number and schedule for delivering messages. In all, 33% (3/9) suggested increasing the number of messages in the late quit stage and 11% (1/9) suggested including a partner to share messages when necessary.

Of the nine participants in the SMS text message smoking cessation program, 56% (5/9) reported having remained smoke-free (not even a puff) during the entire program (until day 30 after quit day); 44% (4/9) reported relapsing on day 2 after quit day. However, of six participants receiving nutritional messages, one (17%) reported having remained smoke-free during the entire program; 33% (2/6) reported relapsing on day 2 after quit day and 50% (3/6) reported relapsing on day 7 after quit day.

## Discussion

This pilot study suggests that a SMS text message cognitive behavioral smoking cessation program would be used by young adults in Lima, Peru. Main findings supporting this conclusion include high recruitment rate and intervention retention rate during the pilot of 93% (14/15), content and technological acceptability, and high self-reported abstinence rate at day 30 after quit day (40%, 6/15; 56%, 5/9 in smoking cessation SMS text message intervention arm).

This pilot demonstrated that it is possible to use low-cost methods (website and social media) to identify and recruit specific populations who may otherwise be difficult to recruit at an on-site location (young adults between ages 18 and 25 who smoke at least four cigarettes daily, etc) [59]. Web-based recruitment strategies are suggested to be associated with better abstinence outcomes than other recruitment strategies [27], probably due to the fact participants are more familiar with technology and technological interventions. Facebook ads may be less cost-effective in Peru than in other countries [60]; however, it may be more effective for recruitment at the national level. Compared with alternatives for pilot studies on smoking cessation, the number of participants recruited was sufficient [59].

Evidence shows that content adaptation is critical for the successful development of mobile behavioral interventions [61]. Relevant information about past experiences and behaviors that contribute to quit characteristics of young adults and Latino populations [33,62-66] was included into the SMS text message cognitive behavioral smoking cessation program content. Participant suggestions were used in designing and modifying the content, syntax, and tone of the SMS text message smoking cessation program content. Participants expressed preferences for messages that contained accurate spelling and grammar (eg, Latin characters), used positive versus negative framing, and were designed to help participants achieve personal goals. These findings coincide with results from other studies [61,67-69]. In addition, adapting the SMS text message cognitive behavioral smoking cessation program content to young adult and Latino populations [33], who have expressed resistance to the use of pharmacotherapy, was an important factor in the development and adaptation of program content to achieve behavioral change [70]. An advantage in Peru for SMS text message health program development and implementation is that there is no cost for receiving SMS text messages unlike other countries, which makes health strategies using SMS text messaging quite accessible.

Some of the preliminary findings from the focus groups and in-depth interviews are similar to findings from systematic reviews and studies in other countries [71,72]: participants perceive the use of e-cigarettes as an accessible, convenient aid in smoking cessation, and less harmful than cigarettes. This alternative can be considered as a resource if evidence (RCTs and population-based studies with precise exposure and safety measures) show that e-cigarettes are at least as effective as NRT in helping young adults smokers to quit [71].

### Limitations

If we want to scale up the pilot, we need to consider strategies to continue and strengthen the high adherence shown in this preliminary results because it may be related to the provision of weekly credit for text messages which allowed participants to provide program feedback (93% overall participation for the entirety of the study; 100% in the intervention arm and 83% in the control arm). Interaction may have increased adherence and response [21]. Additional research is needed to measure adherence without a prepaid card: how much money are participants willing to spend and will they make an effort to continue in the program? Future studies may also help identify whether prepaid cards can help to improve adherence and/or results in long-term studies.

Self-reporting [16,73] (answering yes to “have not smoked even one puff since the quit day”) was used to measure the intervention’s effectiveness; nonetheless, this measure is not the most accurate way to measure abstinence and may produce a high level of negative self-reports among some populations [73,74]. Thus, it would be important to consider biochemical verification of smoking status as an outcome measure in future studies.

In future studies we may consider more accurate forms of measuring abstinence. One approach would be to ask individuals to approach a physical location to collect samples demonstrating abstinence. Another alternative would be to use strips to detect cotinine in the saliva, which can either be mailed by courier or have a photo of the result taken by the participant and sent by mobile phone or email to be read as abstinence confirmation. Portable meters that allow carbon monoxide measurement [75] have already been used in more limited geographical contexts such as universities [76]. This would enable us to request measurement at any time of the day in real time to prevent dose handling. All these measures should be considered for an RCT. Other forms of measuring abstinence include measurement of the saliva cotinine level, carbon monoxide confirmation, and cotinine analysis [73,74]. In addition, in future studies we want to know outcomes in terms of true smoking cessation, which requires increasing follow-up periods to at least 6 months [17,77].

This preliminary data show that a SMS text message cognitive behavioral smoking cessation program can be implemented and should be acceptable for young adults in Lima, Peru, in addition to contributing knowledge about the current evidence gap about appropriateness of a SMS text message smoking cessation program for young adults in a middle-income country with an inactive tobacco control policy [26,27].

The results from our pilot could broaden access to this type of health intervention and be adapted to help other groups quit smoking, such as younger populations, other health conditions (mental, chronic, cardiovascular, neoplastic and infectious diseases), other cultures, and rural areas.

## Appendix

Multimedia Appendix 1 Some of the focus group and in-depth interviews analysis results taken in consideration to adapt characteristics of the smoking cessation SMS text message program.

Multimedia Appendix 2 Examples of SMS text messages.

## Abbreviations

CBT: cognitive behavioral therapy
FTND: Fagerström Test of Nicotine Dependence
MNP: mobile number portability
NRT: nicotine replacement treatment
RCT: randomized controlled trial
SMS: short message service
