# Supplementary figures and images for: Feasibility and Acceptability of a Text Message-Based Smoking Cessation Program for Young Adults in Lima, Peru: Pilot Study
Source: JMIR Mhealth Uhealth. 2017 Aug 4;5(8):e116. doi: 10.2196/mhealth.7532 (PMC5562935; doi:10.2196/mhealth.7532)

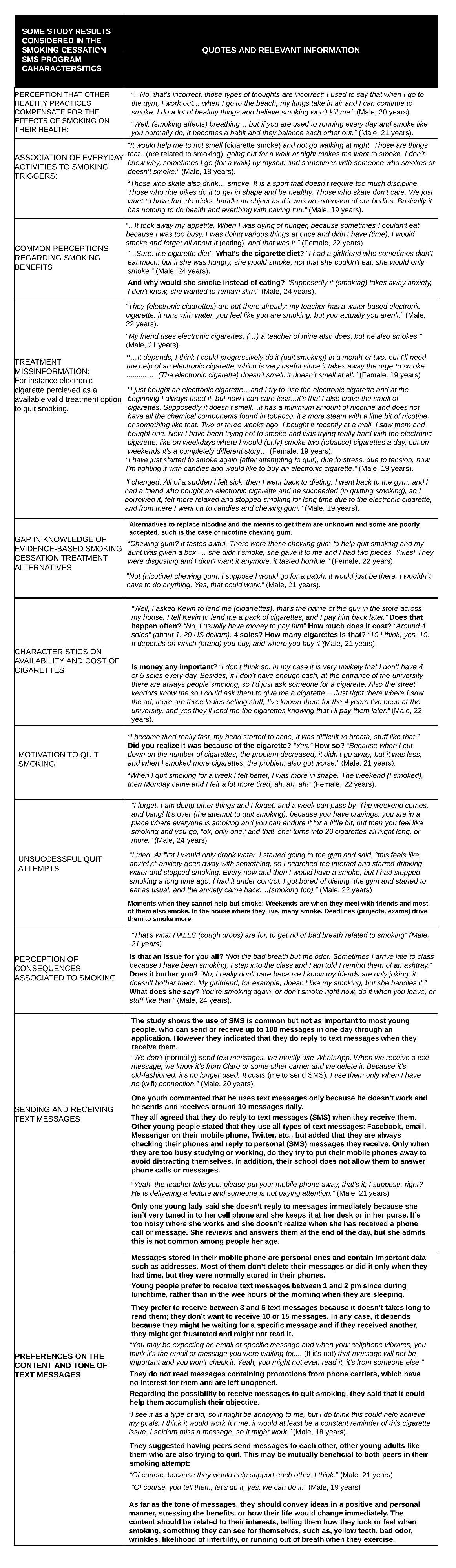

Supplement: Multimedia Appendix 1 [file mhealth_v5i8e116_app1.jpeg]

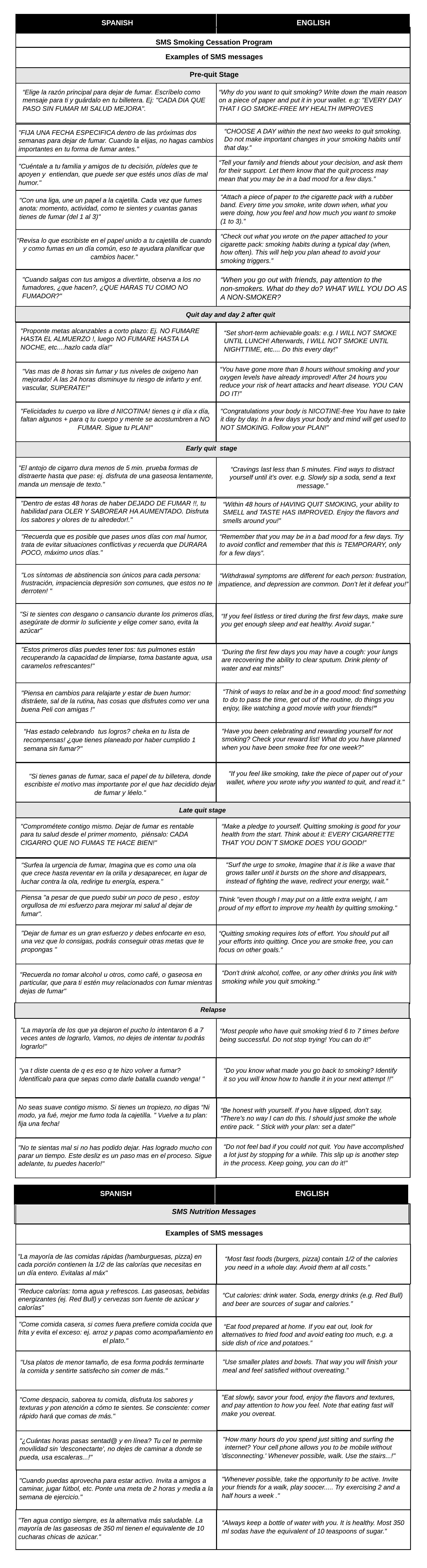

Supplement: Multimedia Appendix 2 [file mhealth_v5i8e116_app2.jpeg]
